# Sensitivity of Cutaneous T-Cell Lymphoma Cells to the Mcl-1 Inhibitor S63845 Correlates with the Lack of Bcl-w Expression

**DOI:** 10.3390/ijms232012471

**Published:** 2022-10-18

**Authors:** Uly Sumarni, Jiaqi Zhu, Tobias Sinnberg, Jürgen Eberle

**Affiliations:** Department of Dermatology, Venerology und Allergology, Skin Cancer Center, Charité—Universitätsmedizin Berlin, 10117 Berlin, Germany

**Keywords:** non-Hodgkin lymphoma, Bcl-2 proteins, BH3 mimetics

## Abstract

Long-term, curative treatment of cutaneous T-cell lymphomas (CTCL) remains a major challenge. Therapy resistance is often based on apoptosis deficiency, and may depend on antiapoptotic Bcl-2 proteins, such as Bcl-2, Bcl-x_L_, Bcl-w and Mcl-1. For their targeting, several antagonists have been generated, which mimic the Bcl-2 homology domain 3 (BH3 mimetics). As dysregulation and overexpression of Mcl-1 has been reported in CTCL, the use of Mcl-1 inhibitors appears as an attractive strategy. Here, we investigated the effects of the selective Mcl-1 inhibitor S63845 in a series of four CTCL cell lines, in comparison to ABT-263 and ABT-737 (inhibitors of Bcl-2, Bcl-x_L_ and Bcl-w). In two cell lines (HH, HuT-78), S63845 resulted in significant apoptosis induction, decrease in cell viability, loss of mitochondrial membrane potential and caspase activation, while two other cell lines (MyLa, SeAx) remained completely resistant. An inverse correlation was found, as S63845-resistant cells were highly sensitive to ABT-263/-737, and S63845-sensitive cells showed only moderate sensitivity to ABTs. Combinations of S63845 and ABT-263 partially yielded synergistic effects. As concerning Bcl-2 protein expression, weaker Mcl-1 expression was found in S63845-resistant MyLa and SeAx, while for Bcl-2 and Bcl-x_L_, the lowest expression was found in the highly sensitive cell line HH. The most striking difference between S63845-resistant and -sensitive cells was identified for Bcl-w, which was exclusively expressed in S63845-resistant cells. Thus, CTCL may be efficiently targeted by BH3 mimetics, providing the right target is preselected, and Bcl-w expression may serve as a suitable marker.

## 1. Introduction

Approximately 5% of non-Hodgkin lymphomas are characterized by primary cutaneous manifestation and clonal proliferation of skin-homing memory T cells, thus forming a distinct group of cutaneous T-cell lymphomas (CTCL). The group encloses mycosis fungoides, Sézary syndrome and CD30^+^ lymphoproliferative disorders [1,2]. In addition to severe effects on patient’s quality of life, about 30% of early-stage CTCL patients undergo disease progression within 10 years by developing tumors as well as by dissemination to lymph nodes, blood and internal organs. Tumor progression is accompanied by reduced 5-year survival rates of only 18% [3]. According to detailed insights into CTCL pathogenesis, several therapeutic strategies have been identified, including TLR7/8 agonist, talimogene laherparepvec, histone deacetylase inhibitors, PI3K inhibitors as well as immune-stimulating antibodies [4]. Nevertheless, long-term, curative treatment remains a major challenge, and the search for new therapeutic approaches continues.

The elimination of tumor cells by apoptosis induction represents a principle goal in cancer therapy; thus, therapy resistance may be frequently explained by apoptosis deficiency [5,6]. As for CTCL, several therapeutic approaches have been related to apoptosis induction, as phototherapy, photopheresis, the retinoid bexarotene and the histone deacetylase inhibitor vorinostat [7,8,9,10].

Apoptosis can be induced through extrinsic and intrinsic pathways. Extrinsic proapoptotic pathways are initiated by death ligand binding to appropriate death receptors and the formation of death-receptor-bound signaling complexes [11]. On the other hand, intrinsic apoptosis pathways can be activated by different types of cellular damage as well as by dysregulation, such as oncogene activation, resulting in loss of mitochondrial membrane potential, mitochondrial permeabilization and release of proapoptotic mitochondrial proteins into the cytoplasm [12,13]. This may be further associated with ROS production [14].

A characteristic hallmark in apoptosis pathways is represented by caspase activation. Thus, death receptor ligation results in initiator caspase-8 activation [11], and mitochondria-released cytochrome c results in the formation of the apoptosome complex and initiator caspase-9 activation [13]. Initiator caspases can activate the major effector caspase-3 in signaling cascades through protease cleavage, which then can cleave a large number of death substrates, with the final result of DNA fragmentation and apoptosis induction [15].

Intrinsic proapoptotic pathways are critically controlled by the family of Bcl-2 proteins, which includes antiapoptotic (e.g., Bcl-2, Mcl-1, Bcl-x_L_ and Bcl-w), proapoptotic BH3-only (e.g., Bid, Bad, Noxa, Puma and Bim) as well as multidomain proapoptotic proteins, such as Bax and Bak. While Bax and Bak are supposed to mediate mitochondrial pores to release proapoptotic factors, they are bound and inhibited by antiapoptotic Bcl-2 proteins, which themselves can be inhibited by the group of BH3-only proteins [16].

As for Mcl-1, accumulating evidence has indicated its critical pro-survival roles in different cell types under physiological as well as malignant conditions. While its expression is tightly regulated at the levels of transcription and protein stability in normal cells, it may be deregulated in tumors [17,18]. Thus, chromosomal amplification, increased mRNA and protein expression were correlated to disease progression and chemotherapy resistance in hematopoietic cancer cells [19,20].

Targeting of antiapoptotic Bcl-2 proteins can be achieved by BH3 mimetics, small molecule inhibitors with structural homology to the Bcl homology domain 3 (BH3). Their binding to the hydrophobic groove of antiapoptotic Bcl-2 proteins prevents their ability to bind other proapoptotic family members [21]. BH3 mimetics with specificity for Bcl-2, Bcl-x_L_ and Bcl-w as ABT-263/navitoclax and ABT-737, as well as mimetics with specificity for Mcl-1 as S63845, have been developed [22,23,24]. ABT-263 represents an orally available analog of ABT-737, which aims to overcome the limited solubility of ABT-737 [22]; ABT-263 has already been tested in clinical trials for patients with hematological malignancies [25].

Dysregulation and overexpression of Mcl-1 was reported in non-Hodgkin and T-cell lymphomas. Mcl-1 expression was also seen in CTCL cell lines as well as in skin lesions from CTCL patients. In biopsies of patients from different CTCL stages, a trend towards increased Mcl-1 expression was found along with an increase in tumor stage [26,27,28]. Thus, the use of selective Mcl-1 inhibitors appears as an attractive therapeutic strategy. In this study, we investigated the effects of S63845 in comparison to ABT-263 and ABT-737 in a series of four CTCL cell lines. The data revealed a subdivision of CTCL cells into two groups with regard to their sensitivity profiles. Mechanisms and causes of resistance were further investigated, resulting in the identification of the selective expression of Bcl-w in S63845-resistant cells.

## 2. Results

### 2.1. CTCL Cell Lines Are Classified as Either Sensitive or Resistant to Mcl-1 Inhibition

**Figure 1:**
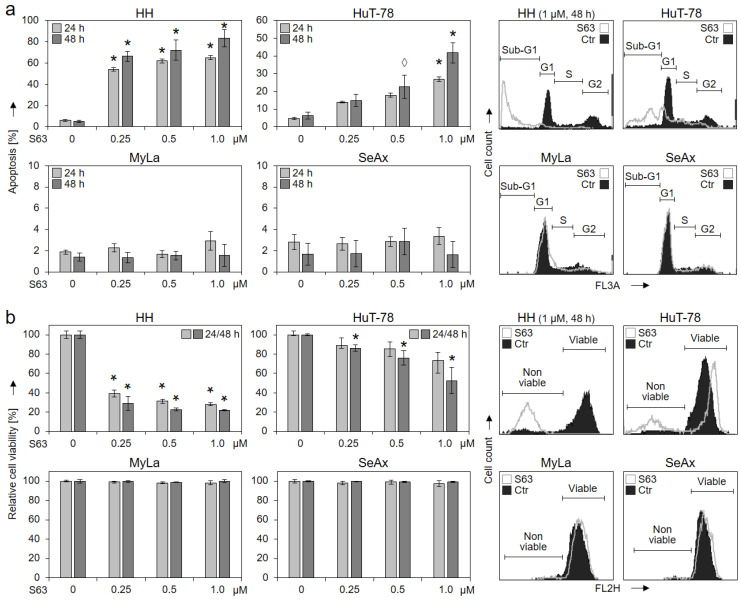
Induced apoptosis and decreased cell viability by S63845 in 2/4 CTCL cell lines. Cell lines HH, HuT-78, MyLa and SeAx were treated with increasing concentrations of S63845 (0.25, 0.5, 1.0 µM). After 24 and 48 h, apoptotic rates were determined by sub-G1 assay (**a**), and rates of cell viability were determined by calcein staining (**b**). Apoptotic rates correspond to percentages of sub-G1 cells (cells with fragmented DNA). Cell viability values were calculated in relation to non-treated controls (0), which were set to 100%. Characteristic histograms of controls (Ctr) and of cells treated for 48 h with S63845 (1 µM) are shown on the right side in overlays. Cell populations in cell cycle phases G1, S, G2 and sub-G1 (**a**) as well as non-viable and viable cells (**b**) are indicated. Mean values of triplicates ± SD of representative experiments are shown. At least three independent experiments were performed, which showed highly comparable results. Statistical significance was calculated from the mean values of independent experiments and is shown for treated cells vs. controls (*, *p* < 0.05). For some values, a statistical trend is also indicated (◊, *p* < 0.1).

For evaluation of the possible therapeutic potential of selective Mcl-1 inhibitors in CTCL, four representative cell lines (HH, HuT-78, MyLa and SeAx) were treated with increasing concentrations of S63845 (0.25, 0.5, 1.0 µM). Apoptotic rates were determined by sub-G1 assay, and cell viability was determined by calcein staining at 24 h and 48 h. While two cell lines (HH and HuT-78) showed strong response to S63845, cell lines MyLa and SeAx were completely resistant. A dose-dependent increase in apoptotic rates was found in sensitive cells: 66%/72%/83% (in HH) and 15%/23%/42% (HuT-78) for 0.25/0.5/1.0 µM S63845 at 48 h, respectively (Figure 1a). In parallel, cell viability was strongly decreased to 22% (HH) and to 53% (HuT-78; 1 µM, 48 h; Figure 1b).

To determine EC50 values for S63845, additional experiments were performed for the four cell lines, including concentrations between 10 nM and 2 µM of S63845. Cell viability (Supplementary Figure S1) and apoptosis (Figure S2) were determined after 24 h and 48 h. As for HH at 48 h, 22 nM was determined for 50% loss of cell viability and 95 nM for 50% induced apoptosis. As for HuT-78 at 48 h, 110 nM was determined for 50% loss of cell viability and at 1.4 µM for 50% induced apoptosis. As for the resistant cell lines SeAx and MyLa, even the highest concentration (2 µM) was not sufficient; thus EC50 was >2 µM (Figures S1 and S2).

### 2.2. Activation of Apoptosis-Related Pathways by S63845

**Figure 2:**
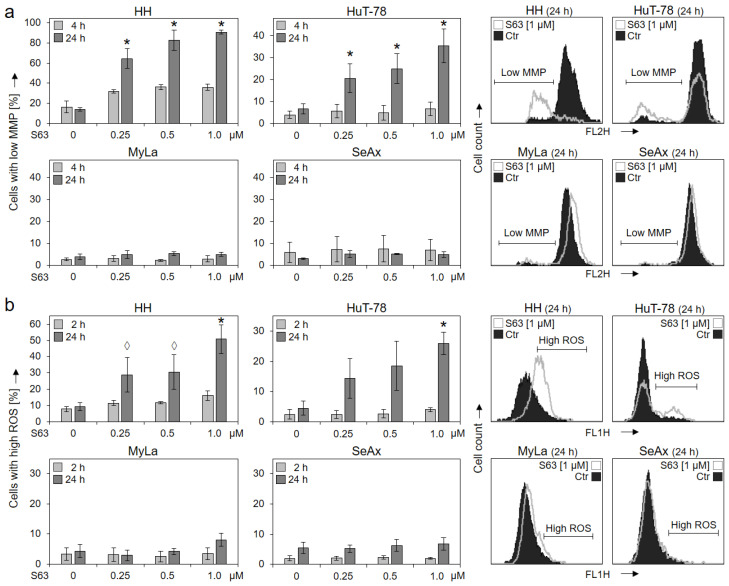
Loss of MMP and enhanced ROS levels in sensitive cells. Cell lines HH, HuT-78, MyLa and SeAx were treated with increasing concentrations of S63845 (0.25, 0.5, 1.0 µM). Changes of mitochondrial membrane potential (MMP, TMRM^+^ staining) were determined at 4 h and at 24 h of treatment (**a**); levels of reactive oxygen species (ROS, H_2_DCF-DA staining) were determined at 2 h and at 24 h of treatment (**b**). Representative histograms (overlays of cells treated for 24 h with 1 µM S63845 vs. controls, Ctr) are given on the right side. Mean values of triplicates ± SD of representative experiments are shown here; three independent experiments were performed for 24 h, which had highly comparable results; two experiments were performed for 2 h/4 h (each one with triplicates). Statistical significance (24 h) was calculated from the mean values of independent experiments and is shown for treated cells vs. controls (*, *p* < 0.05). For some values, a statistical trend is also indicated (◊, *p* < 0.1).

Loss of mitochondrial membrane potential (MMP), which represents a key trigger of intrinsic mitochondrial apoptosis pathways, was monitored at 4 h and at 24 h of treatment. Significant and dose-dependent loss of MMP was found at 24 h in response to treatments with 0.25, 0.5 and 1.0 µM of S63845 in HH (65%, 83% and 91%) as well as in HuT-78 (21%, 25% and 35%), while MyLa and SeAx showed no response (Figure 2a).

Increased levels of reactive oxygen species (ROS) may result from mitochondrial activation and mitochondrial leakage and may activate further cell death pathways in cancer cells [29]. Again, HH and HuT-78 showed increased ROS after 24 h in 51% and 26% of cells (c = 1 µM), respectively, while no clear response was seen in MyLa and SeAx (max. 8% ± 2%; Figure 2b).

**Figure 3:**
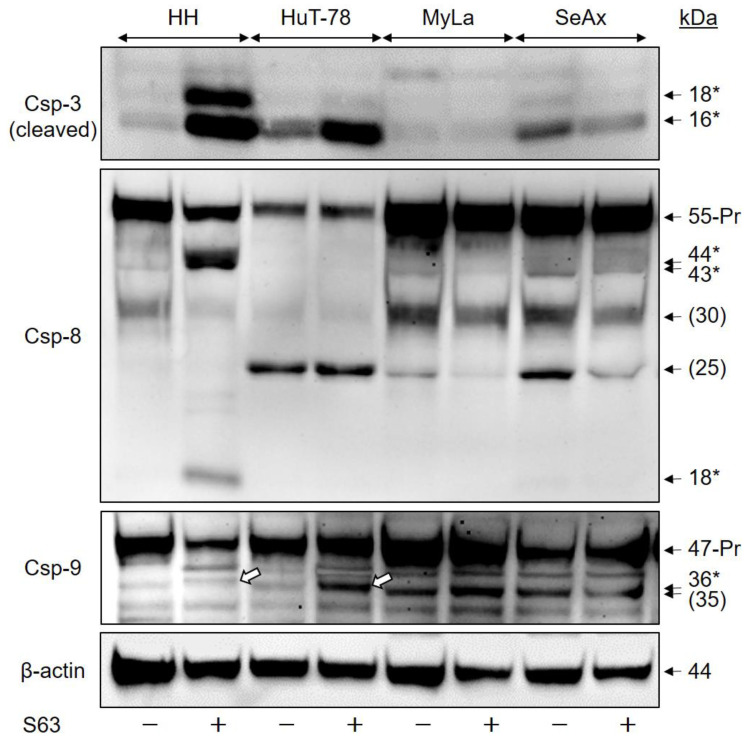
Caspase activation by S63845. CTCL cell lines were treated with 1 µM S63845, and activation of caspase cascade was determined at 48 h in Western blotting by analysis of the induced, specific cleavage products, as compared to DMSO-treated controls. Each 30 µg of proteins were loaded per lane. Blots were probed with antibodies for the cleaved forms of the major proapoptotic effector caspase-3 (cleavage products: 18, 16 kDa), for caspase-8, the initiator caspase of extrinsic pathways (proform: 55 kDa; cleavage products, 44, 43, 18 kDa) as well as for caspase-9, the initiator caspase of intrinsic pathways (proform, 47 kDa; cleavage product: 36 kDa). Specific cleavage products are indicated by (*), the cleavage products of caspase-9 (36 kDa) are further indicated by white arrows in the figure). Protein bands at 30 and 25 kDa (caspase 8) as well as 35 kDa (caspase-9) were not specified and do not reflect caspase activation (put in brackets). The housekeeping protein β-actin (44 kDa) was used as loading control. Two independent series of protein extracts and Western blotting experiments revealed highly comparable results.

Activation of caspase cascades via caspase-8 to caspase-3 or via caspase-9 to caspase-3 are central in apoptosis regulation. Indicating the contribution of caspases, we found strong activation of effector caspase-3 in the two sensitive cell lines, seen in Western blots by the major activated cleavage product of 16 kDa. In contrast, no induction of the 16 kDa form was seen by S63845 treatment in MyLa and SeAx, although weakly expressed in non-treated SeAx cells (Figure 3).

In HH, caspase-3 activation correlated with activation of caspase-8, the initiator caspase of extrinsic apoptosis pathways, seen by the cleavage products of 44, 43 and 18 kDa. Furthermore, caspase-9, the initiator caspase of the intrinsic apoptosis pathway, was activated in HuT-78, as seen by a cleavage product of 36 kDa. A non-specified, close protein band of 35 kDa was responsive to caspase-9 antibody. As this 35 kDa band was also seen in non-treated cells, it may thus not be related with caspase-9 activation (Figure 3). Thus, S63845 treatment was able to trigger both extrinsic and intrinsic caspase cascades in different sensitive cell lines, which was excluded in resistant cells.

Caspase activation was quantified by densitometric analysis of the activated processing products of 16 kDa for caspase-3, 18 kDa for caspase-8 as well as 36 kDa for caspase-9. After normalization by the respective β-actin values, induction factors were calculated from two independent experiments, and median values were formed, resulting in median induction factors. Thus, the median induction factors for caspase-3 were 16 (HH) and 4 (HuT-78), while the factors were 1.2 and 0.9 for MyLa and SeAx, respectively. The median induction factors for caspase-8 were 28 (HH), whereas there was no significant caspase-8 activation in HuT-78, MyLa and SeAx. The caspase-9 median induction factor for HuT-78 was determined as 2.6, whereas it was 1.9 (HH), 1.2 (MyLa) and 0.9 (SeAx).

### 2.3. High Sensitivity of MyLa and SeAx to ABT-263 and ABT-737

**Figure 4:**
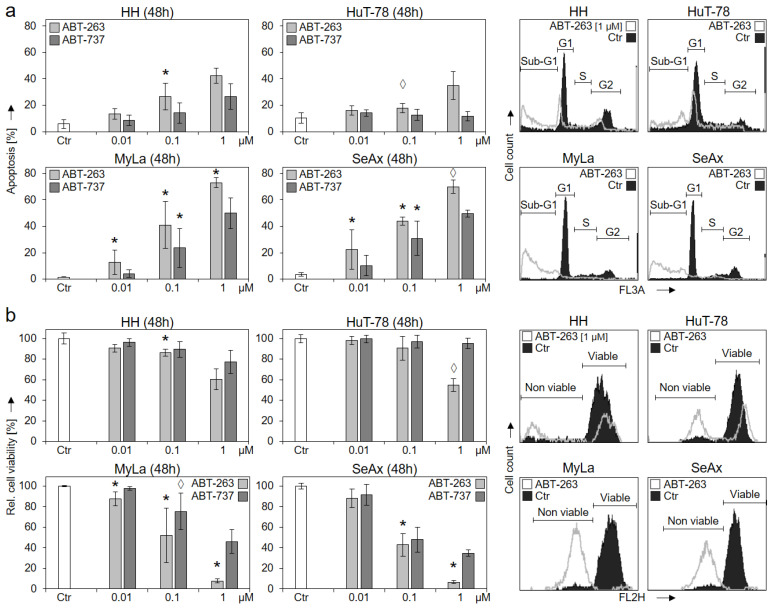
Strong response of S63845-resistant cells to ABT-263 and ABT-737. CTCL cell lines were treated with increasing concentrations (0.01, 0.1, 1 µM) of ABT-263 (light grey) and ABT-737 (dark grey). Apoptotic rates (**a**) were determined by sub-G1 assay, and cell viability rates (**b**) were determined by calcein staining at 48 h. Apoptotic rates correspond to percentages of sub-G1 cells (cells with fragmented DNA). Cell viability values were calculated in relation to non-treated controls (Ctr), which were set to 100%. Example histograms of controls and of cells treated with 1 µM ABT-263 are shown on the right side in overlays. Cell populations in cell cycle phases G1, S, G2 and sub-G1 (**a**) as well as non-viable and viable cells (**b**) are indicated. Mean values of triplicates ± SD of representative experiments are shown. At least three independent experiments (each one with triplicates) were performed for ABT-263 (0.01, 0.1 and 1 µM) as well as for ABT-737 (0.1 µM), which showed highly comparable results. Statistical significance was calculated from the mean values of independent experiments, and is shown for treated cells vs. controls (*, *p* < 0.05). For some values, a statistical trend was also indicated (◊, *p* < 0.1). For ABT-737 (0.01 and 1 µM) only two independent experiments (each one with triplicates) were performed. Thus, here, median values are given, and no statistical significance is indicated.

The comparison with ABT-263 and ABT-737, which target the antiapoptotic Bcl-2 proteins Bcl-2, Bcl-x_L_ and Bcl-w, revealed an inverse correlation. Thus, treatment with increasing concentrations (0.01, 0.1 and 1 µM) for 48 h showed particularly high and dose-dependent sensitivity of the S63845-resistant cell lines MyLa and SeAx. For 1 µM ABT-263, apoptosis induction in MyLa and SeAx was at 73% and 70%, respectively, while only 43% and 35% apoptosis was induced in HH and HuT-78 (Figure 4a).

Largely comparable, cell viability was strongly decreased in MyLa and SeAx by 1 µM ABT-263 to 8% and 7% of controls, while the effect was only 60% and 55% in HH and HuT-78, respectively (Figure 1b). It may thus be concluded that the survival of HH and HuT-78 was particularly dependent on Mcl-1, while the survival of MyLa and SeAx was based on the other Bcl-2 proteins. As determined from at least three independent experiments, the response of MyLa and SeAx to ABT-263 (0.1 and 1 µM) in terms of apoptosis induction and loss of cell viability was significantly higher than in HH and HuT-78 (*p* < 0.01).

### 2.4. Effects of Bcl-2 Protein Antagonists Are Mutually Enhanced in Combinations

**Figure 5:**
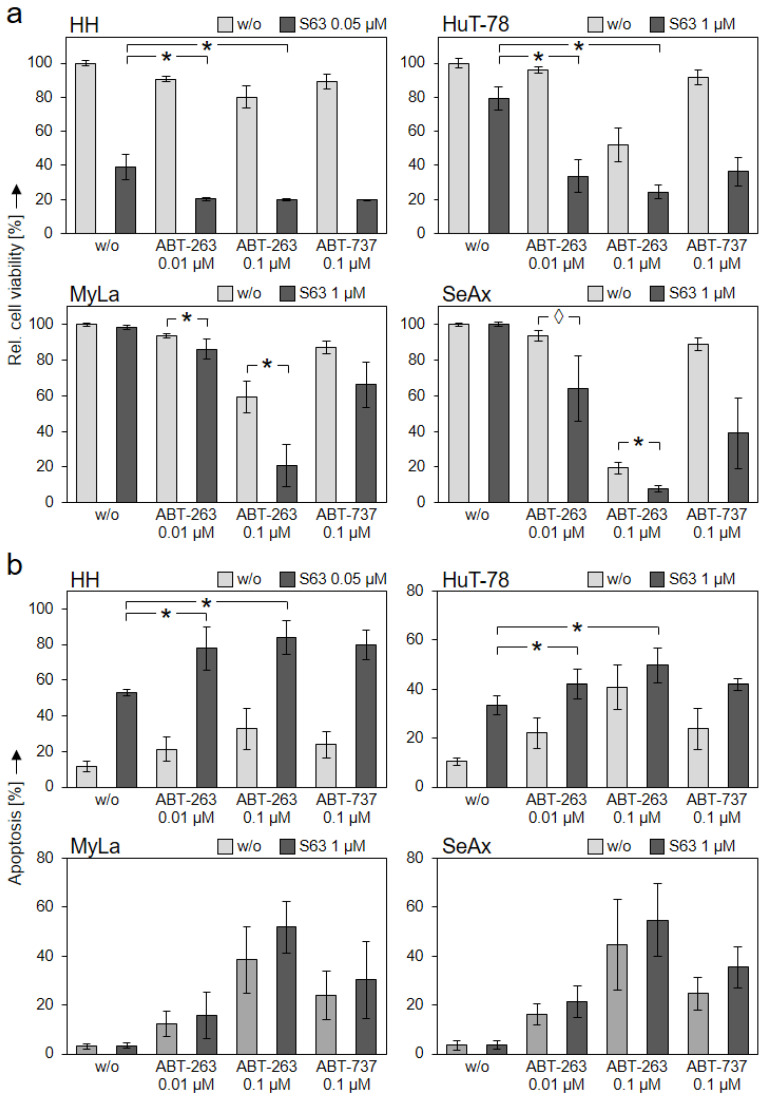
Further enhanced effects by antagonist combinations. CTCL cell lines were treated with S63845 (HH: 0.05 µM; HuT-78, MyLa, SeAx: 1 µM, dark bars), with ABT-263 (0.01 and 0.1 µM), with ABT-737 (0.1 µM) as well as with respective combinations. Cell viability (calcein staining, (**a**)) and apoptotic rates (sub-G1 assay, (**b**)) were determined after 48 h. Cell viability values were calculated in relation to non-treated controls, which were set to 100%; apoptotic rates correspond to percentages of sub-G1 cells (cells with fragmented DNA). Mean values of triplicates ± SD of representative experiments are shown. At least three independent experiments (each one with triplicate values) were performed for ABT-263 combinations, which showed highly comparable results. For ABT-737 combinations, two independent experiments (each one with triplicate values) revealed comparable results. Statistical significance (ABT-263) was calculated from the mean values of independent experiments, and is shown for cells treated with combinations vs. single treatments (*, *p* < 0.05). For some values, a statistical trend is also indicated (◊, *p* < 0.1).

We investigated whether CTCL cells may be even better targeted by the combination of different BH3 mimetics. Lower concentrations of ABTs (down to 0.1 µM) were applied in all cell lines, and only 0.05 µM of S63845 was applied in HH, to better recognize any mutual enhancement. Good combination effects were obtained at the level of cell viability. Thus, in S63845-sensitive HH and HuT-78, effects of S63845 were further enhanced by ABT-263 and ABT-737. In HH, cell viability further decreased from 39% (0.05 µM S63845) to 20% (combination with 0.1 µM ABT-263) and to 19% (combination with 0.1 µM ABT-737). In HuT-78, cell viability decreased from 79% (1 µM S63845) to 24% (combination with 0.1 µM ABT-263) and to 36% (0.1 µM ABT-737) (Figure 5a).

Although MyLa and SeAx were not responsive to S63845 alone, the cell viability already decreased in response to ABT-263 and ABT-737 was further reduced in combinations with S63845. Thus, cell viability in MyLa was reduced from 59% (0.1 µM ABT-263) to 21% in combination with 1 µM S63845, and it was reduced from 87% (0.1 µM ABT-737) to 66% in combination. Similarly in SeAx, cell viability was reduced from 19% (0.1 µM ABT-263) to 8% in combination with 1 µM S63845, and it was reduced from 89% (0.1 µM ABT-737) to 39% in combination (Figure 5a). At the level of apoptosis induction, however, the effects of combinations were less pronounced, as compared to single treatments (Figure 5b).

For addressing the question of whether these enhancements were synergistic, a large series of combination experiments with increasing concentrations of the antagonists S63845 and ABT-263 were performed (Supplementary Figure S3). Using the web application SyngeryFinder 3.0 created by Ianevski et al. [30], the enhancement of viability reduction and apoptosis induction in HH and HuT-78 cells was calculated as largely synergistic (δ score > 10). Over larger concentration ranges, the δ score was >40 in HH, and 10–40 in HuT-78, respectively. On the other hand, for cell viability in MyLa and SeAx, a synergistic relationship with δ > 10 was seen only for a limited number of combined concentrations. Additionally, for the induction of apoptosis, the δ scores were between −10 and 10 in these cells, suggesting an additive effect (Supplementary Figure S4).

The effects of Bcl-2 protein antagonists were, however, not restricted to CTCL cells, as shown in experiments with freshly isolated PBMCs. Thus, four independent PBMC preparations cultured in the same growth medium as CTCL cells and treated for 48 h with S63845 showed comparably reduced cell viability and induced apoptosis as sensitive CTCL cells. Thus, cell viability was reduced at 48 h to 31%, 17%, 6% and 4%, and apoptosis was induced to 54%, 49%, 43% and 41% (S63845, 1 µM). Similar effects were seen for ABT-263 (1 µM) and for ABT-737 (1 µM) (Supplementary Figure S5).

### 2.5. Expression of Bcl-w Correlates with Variant Sensitivities

To obtain a better understanding of the possible causes of variation in the sensitivity of CTCL cell lines to the different Bcl-2 protein antagonists, expression levels of four major antiapoptotic Bcl-2 proteins (Mcl-1, Bcl-x_L_, Bcl-2 and Bcl-w) were investigated by Western blotting. As suppression of Mcl-1 activity might affect the protein levels, we included CTCL samples before and after S63845 treatment (48 h, 1 µM).

**Figure 6:**
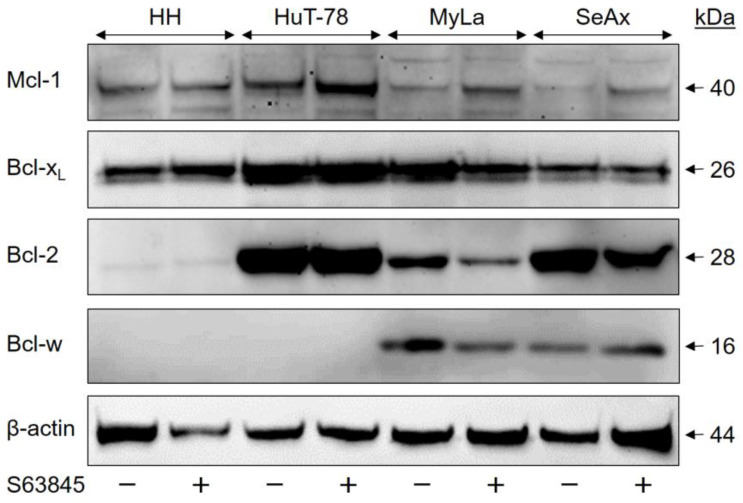
Variant expression of the Bcl-2 family proteins in CTCL cells. CTCL cell lines treated for 48 h with S63845 (1 µM, +) were compared to control cells (DMSO, −). In each lane, 30 µg of proteins were loaded. Blots were probed with antibodies for Mcl-1 (40 kDa), Bcl-x_L_ (26 kDa), Bcl-2 (28 kDa) and Bcl-w (16 kDa). The housekeeping protein β-actin (44 kDa) was used as a loading control. Three independent series of protein extracts and Western blotting experiments revealed highly comparable results.

Mcl-1 protein was generally expressed in all four CTCL cell lines (Figure 6). However, its expression was weaker in S63845-resistant CTCL cell lines than in sensitive cells, as calculated after densitometric quantification and normalization by β-actin from three independent WB experiments (*p* < 0.05). When the mean expression in HH was set to 100% (±34%), it was 102% ± 57% in HuT-78, but only 61 ± 43% and 58 ± 34% in MyLa and SeAx, respectively. In contrast, both Bcl-2 and Bcl-x_L_ were most weakly expressed in HH (Figure 6). As for Bcl-x_L_, quantitative analysis of three independent experiments revealed a relative expression in HH of 27% ± 9%, as compared to HuT-78 (100% ± 34%), MyLa (75% ± 24%) and SeAx (71% ± 57%; *p* < 0.01). The expression of Bcl-2 was quantified from two independent experiments, but as there was no difference between (−S63845) and (+S63845) samples; all four values were used for analysis. Thus, expression of Bcl-2 was 6% ± 5% in HH, as compared to HuT-78 (100% ± 7%), MyLa (27% ± 8%) and SeAx (74% ± 20%). The most striking difference between S63845-resistant and -sensitive cells was identified at the level of Bcl-w expression. This protein was completely lacking in HH and HuT-78 (<1%), while it was significantly expressed in MyLa (100% ± 20%) and SeAx (97% ± 26%), as determined from three independent experiments (Figure 6).

These data support a hypothesis according to which apoptosis resistance in MyLa and SeAx is particularly based on Bcl-w, while Mcl-1 is important for HH and HuT-78. Whereas in HuT-78, MyLa and SeAx, the antiapoptotic potential of Bcl-x_L_ and Bcl-2 can diminish the effects of the Mcl-1 inhibitor S63845, the cell line HH was particularly dependent on Mcl-1, and thus, S63845 showed the strongest effects.

## 3. Discussion

Activation of proapoptotic signaling pathways is an important goal in cancer therapy, which is often blocked by antiapoptotic Bcl-2 proteins. The targeting of Bcl-2 proteins thus appears as a promising strategy. A number of BH3 mimetics have been developed, which mimic the activity of proapoptotic BH3-only proteins and bind to the antiapoptotic Bcl-2 counterparts [21]. In this way, the blockage of apoptosis may be overcome, leading to either direct apoptosis induction or increased therapy sensitivity. Among the first BH3 mimetics, ABT-737 and ABT-263 were shown to inhibit Bcl-2, Bcl-x_L_ and Bcl-w [22,23].

As for Mcl-1, its important roles have been reported in hematopoietic cancer cells of different types, such as multiple myeloma (MM) [31], diffuse large B-cell lymphoma (DLBCL) [19], mantle cell lymphoma (MCL) [32] as well as in non-Hodgkin and T-cell lymphomas [26,27]. The development of BH3 mimetics with specificity for Mcl-1, e.g., S63845, enabled selective Mcl-1 targeting, which may also be used in therapeutic approaches [24]. When applying S63845 in four CTCL cell lines, we observed a clear division between two completely resistant cell lines and two sensitive cell lines, the latter characterized by induced apoptosis and decreased cell viability. The response was not correlated to the cells’ origin, being derived either from MF or Sézary patients.

In contrast to the here-reported two, out of four, resistant CTCL cell lines, the vast majority of other lymphoma cell lines have previously been reported as sensitive. For example, apoptosis induction by S63845 was seen in 13 cell lines of DLBCL, in four out of five cell lines of Burkitt lymphoma (BL) and in 11 cell lines of primary effusion lymphoma [33,34]. As concerning cell viability, all investigated cell lines of AML and 23/25 cell lines of multiple myeloma showed IC50 values of <1 µM. For chronic myeloid leukemia (CML), all of the five investigated cell lines were reported as resistant to S63845 [24,35]. Thus, the question was addressed, which factors decide the variation in the sensitivity of CTCL cells.

Apoptosis can be mediated through extrinsic and intrinsic caspase cascades via caspase-8 and caspase-9, respectively, which finally results in the activation of the major effector caspase-3 [15]. Caspase activation appears to be of particular importance for apoptosis induction in CTCL cells, as seen in response to different treatments, e.g., histone deacetylase inhibitors, non-steroidal anti-inflammatory drugs or inhibitors for protein kinase C delta [9,36,37]. Additionally, in response to S63845, significant caspase-3 activation was evident in sensitive CTCL cells, caspase-8 processing was seen in HH, and caspase-9 processing was seen in HuT-78, suggesting that both extrinsic and intrinsic caspase pathways may be activated by S63845. Caspase-3 activation in response to S63845 was also reported in DLBCL, Burkitt lymphoma and AML cells [33,35,38].

Loss of mitochondrial membrane potential represents an essential step in intrinsic apoptosis pathways [13], which may be further associated with ROS production [14]. In response to S63845, significant loss of MMP and ROS production was observed at 24 h in sensitive CTCL cell lines, suggesting the contribution of these pathways. Loss of MMP in response to S63845 was also reported in DLBCL and AML cells [35,38]. To date, ROS have not been investigated in most lymphoma studies, but their induction was also found in two AML cell lines at 24 h in response to S63845 [39]. Thus, as resistant CTCL cell lines were completely lacking caspase activation as well as loss of MMP and ROS production in response to S63845, the causes of their resistance had to be sought more above in the pathway.

Our comparison with two alternative BH3 mimetics (ABT-263 and ABT-737), which target Bcl-2, Bcl-x_L_ and Bcl-w, revealed a particular inverse correlation. Namely, high sensitivity to these ABTs was found in S63845-resistant MyLa and SeAx cells, while S63845-sensitive HH and HuT-78 showed only moderate sensitivity to ABTs. It appears that CTCL cells can be targeted, in principle, by BH3 mimetics, providing the right ones are chosen. Efficacy of ABT-737 was also reported in several other lymphoid cancer cells [40], while resistance was also reported in cell lines of, for example, CLL, ALL [41], MCL [42] and multiple myeloma [43]. As for ABT-263, both sensitive and resistant cell lines of different hematological tumors have been reported [22,23].

Thus, CTCL cell lines differentiated in two groups characterized either by high sensitivity to S63845 and only moderate sensitivity to ABTs, or by S63845 resistance and high sensitivity to ABTs. We aimed to explain these varying responses by differences in the expression of antiapoptotic Bcl-2 proteins. Via Western blot analysis, we showed that Mcl-1 protein was more weakly expressed in S63845-resistant CTCL cell lines, whereas Bcl-2 and Bcl-x_L_ expression was weakest in the highly sensitive cell line HH. The most striking difference was identified for Bcl-w, which was exclusively expressed in CTCL cells with S63845 resistance and high sensitivity to ABT-263 and -737. These findings suggest that survival of CTCL cells may be critically controlled by the different antiapoptotic Bcl-2 proteins. Once Bcl-w is expressed, it is strong enough to prevent apoptosis induction through Mcl-1 inhibition, but exhibits high sensitivity to ABT-263 and -737. Vice versa, when Bcl-w is lacking, CTCL survival particularly depends on Mcl-1, resulting in a high S63845 sensitivity and reduced sensitivity to ABT-263 and -737. In this setting, Bcl-2 and Bcl-x_L_ may have contributory roles. When they are weakly expressed, the sensitivity to Mcl-1 inhibition further increases.

Important roles of Bcl-w have also been described in B-cell lymphoma, Burkitt lymphoma and DLBCL [44]. However, the downregulation of Bcl-w in DLBCL cell lines did not sensitize them to S63845 [45]. Another inverse correlation was reported for Bcl-x_L_ in AML, CML and DLBCL cells, where S63845 resistance correlated with high Bcl-x_L_ expression, suggesting that in these cells, Bcl-x_L_ may functionally compensate for the inhibition of Mcl-1 [24,38].

BH3 mimetics represent promising candidates for clinical application in cancer patients [21]. In this study, we also found high sensitivity of isolated PBMCs to S63845 as well as to ABT-263 and ABT-737, in terms of apoptosis induction and loss of cell viability. However, it remains to be clarified, whether the response of cultured PBMCs ex vivo may reflect a similar response in the in vivo situation. Cultured normal cells in growth medium may lack essential survival signals that may be provided in the tissue. Finally, animal experiments and the clinical testing may answer this question. So far, S63845 has been described as well tolerated by mice, with no significant weight loss observed [24], and it is currently in a phase I clinical trial [46]. Also, suitable safety profiles were reported for ABT-263 in phase I/II clinical trials for hematological malignancies [47].

In terms of clinical efficacy, ABT-263 has reportedly shown efficacy in combination with another BH3 mimetic (ABT-199) in a phase I trial with patients with ALL or lymphoblastic lymphoma [48]. For targeting Mcl-1, two Mcl-1 inhibitors are currently being evaluated in ongoing phase I/II clinical trials for different hematological cancers, namely PRT1419 [49] and S64315 (MIK665). The latter is chemically related to S63845, and shows comparable activity [50]. Open questions of dosing and the safety profile resulted in the termination of some previous studies. Currently, S64315 is in a phase I clinical trial to evaluate the maximal tolerated dose and the recommended dose for expansion. Further studies are planned to evaluate the tolerability, safety and antitumor activity of S64315 in different lymphoid tumors [46]. As Mcl-1 is a promising target in cancer treatment, there is much hope that Mcl-1-based strategies may also be well tolerated.

Taken together, our findings indicate promising responses of CTCL cells to BH3 mimetics. CTCL cell lines were either highly sensitive to the inhibition of Mcl-1 (S63845) or to the inhibition of Bcl-2/Bcl-x_L_/Bcl-w (ABT-263, ABT-737). Sensitivity to S63845 was correlated with diminished expression of Bcl-2 and Bcl-x_L_ and in particular with a complete lack of Bcl-w expression. These findings shed more light on the particular roles of antiapoptotic Bcl-2 proteins in apoptosis control in CTCL cells. Furthermore, therapeutic strategies may be considered that are either based on Mcl-1 inhibitors or on ABT-263, or a combination of both. Expression levels of Bcl-w in tumor T cells may be used as a biomarker to identify the right targets.

## 4. Materials and Methods

### 4.1. Cell Culture and Treatment

Four representative CTCL cell lines were used in this study: MyLa derived from a plaque biopsy of a patient with MF [51]; SeAx [52] and HuT-78 [53] derived from peripheral blood mononuclear cells of patients with Sézary syndrome; and HH (ATCC, Manassas, VA, USA; CRL2105) derived from peripheral blood of a patient with aggressive CTCL [54]. Cells were maintained at 37 °C and 5% CO_2_ in RPMI 1640 growth medium (Life Technologies, Darmstadt, Germany) supplemented with fetal calf serum (FCS, 10%), L-glutamine (600 μM) and antibiotics. Cells were generally passaged twice a week.

For the different assays, 5 × 10^4^ cells were seeded per well in 24-well plates. Cells were treated with 0.01–2 µM S63845 (CAS# 1799633-27-4, Hölzel Diagnostika, Cologne, Germany), 0.01–1 µM ABT-263 (CAS# 923564-51-6, Biozol, Eching, Germany) and 0.01–1 µM ABT-737 (CAS# 852808-04-9, Biozol). Control cells received the solvent DMSO in the same concentration as used for treatment (max. 0.4%).

For control, human peripheral blood mononuclear cells (PBMCs) were isolated from full blood of healthy, voluntary donors (20 mL). All donors were informed about the use of their material. For reasons of data protection, samples were anonymized and randomized. PBMCs were isolated by a Ficoll 400 density gradient (Biocoll, Bio&SELL, Feucht/Nürnberg, Germany). Cells were washed in cold PBS, and healthy cells were counted after trypan blue staining. For assays, PBMCs were seeded the same day in 24-well plates (100,000 cells/well). Treatment with Bcl-2 protein agonists started after 24 h, and assays were performed after 48 h of treatment.

### 4.2. Determination of Apoptosis, Cytotoxicity, Cell Viability and Cell Proliferation

Quantification of apoptosis was performed by cell cycle analysis and sub-G1 assay. Harvested cells were lysed in hypotonic buffer containing sodium citrate (0.1%), Triton-X 100 (0.1%) and propidium iodide (PI, Sigma-Aldrich, St. Louis, MO, USA, 40 µg/mL). Thus, cells were lysed, and isolated cell nuclei were stained for at least 1 h with propidium iodide at 4 °C. Cells in G1, G2 and S phases as well as sub-G1 cells were quantified by flow cytometry at FL3A with a FACSCalibur (BD Bioscience, Bedford, MA, USA). Due to the washing out of small DNA fragments, nuclei with less DNA than G1 (sub-G1) correspond to apoptotic cells.

Cell viability was determined by staining cells with calcein-AM (PromoCell, Heidelberg, Germany), which is converted through intracellular esterase activity in viable cells to the green fluorescent calcein. Cells, grown and treated in 24-well plates, were harvested and stained for 1 h with 0.5 µM calcein-AM at 37 °C. After labeling, cells were washed with PBS and measured by flow cytometry (FL2H).

### 4.3. Determination of Mitochondrial Membrane Potential and Reactive Oxygen Species (ROS)

The potential across the mitochondrial membrane was quantified by staining cells with the fluorescent dye TMRM^+^ (tetramethylrhodamine methyl ester, Sigma-Aldrich). Cells, grown and treated in 24-well plates, were harvested and stained for 20 min at 37 °C in 1 µM TMRM^+^. After washing twice with PBS, cell staining was quantified by flow cytometry (FL2H).

For the determination of intracellular ROS levels, cells grown in 24-well plates were preincubated for 1 h with the fluorescent dye H_2_DCFDA (2’,7’-dichlorofluorescin diacetate, D-399, Thermo Fisher Scientific, Hennigsdorf, Germany, 10 µM), before starting treatment with effectors. After 2–24 h of treatment, cells were harvested, washed two times with PBS and analyzed by flow cytometry (FL1H). As positive control, H_2_O_2_ (1 mM) was applied for 1 h.

### 4.4. Western Blotting

For Western blotting, total protein extracts were obtained in cell lysis buffer containing 150 mM NaCl, 1 mM EDTA, 1% NP-40, 50 mM Tris (pH 8.0) as well as phosphatase and protease inhibitors. Protein concentrations were determined by BCA staining (#23225, Thermo Fisher Scientific, Hennigsdorf, Germany) as compared to BSA concentration standard. Following SDS polyacrylamide gel electrophoresis, proteins were blotted on nitrocellulose membranes, as described previously [55].

Several primary antibodies were derived from Cell Signaling Technology (Danvers, MA, USA): caspase-3 (9662, rabbit, 1:1000), caspase-8 (9746, mouse, 1:1000), caspase-9 (9502, rabbit, 1:1000), Mcl-1 (4572, rabbit, 1:1000), Bcl-w (2724, rabbit, 1:1000) and Bcl-2 (2872, rabbit, 1:1000). Other primary antibodies were derived from Santa Cruz Biotech (Dallas, TX, USA): Bcl-x_L_ (sc-8392, mouse, 1:1000) and β-actin (sc-47778, mouse, 1:1000). As secondary antibodies, peroxidase-labeled goat anti-rabbit and goat anti-mouse were used (Dako, Hamburg, Germany; 1:5000).

### 4.5. Statistical Analyses

All analyses were proven by two to five independent experiments. Each independent experiment itself consisted of triplicate values (three wells that were seeded, treated and analyzed individually). For determination of statistical significance by Student’s *t*-test, only the mean values of the independent experiments were used (3–5 values). Statistical significance is indicated by asterisks in the presented figures (*, *p* < 0.05).

For Western blots, two experiments (caspases) or three experiments (Bcl-2 proteins) were performed, which derived from independent series of protein extracts. All signals were quantified by densitometric analysis and were normalized by the respective β-actin values. For the Bcl-2 proteins with three independent experiments, statistical evaluations were performed.

As concerning the identification of synergistic effects in combination treatments with S63845 and ABT-263, the program SyngeryFinder 3.0 was used [30]. In particular, the Bliss scoring method was applied, and delta scores (δ) ≥10 were considered as synergistic, whereas scores between −10 and 10 were considered as additive.

## Data Availability

Not applicable.

## Supplementary Materials

The following supporting information can be downloaded at: https://www.mdpi.com/article/10.3390/ijms232012471/s1.
