# Supplementary material for: Sensitivity of Cutaneous T-Cell Lymphoma Cells to the Mcl-1 Inhibitor S63845 Correlates with the Lack of Bcl-w Expression
Source: Int J Mol Sci. 2022 Oct 18;23(20):12471. doi: 10.3390/ijms232012471 (PMC9604298; doi:10.3390/ijms232012471)
Supplement: Supplementary file 1 [file ijms-23-12471-s001.zip › CTCL S63 - Figure S1 (dose response - viability) - 02.pptx]

## Slide 1
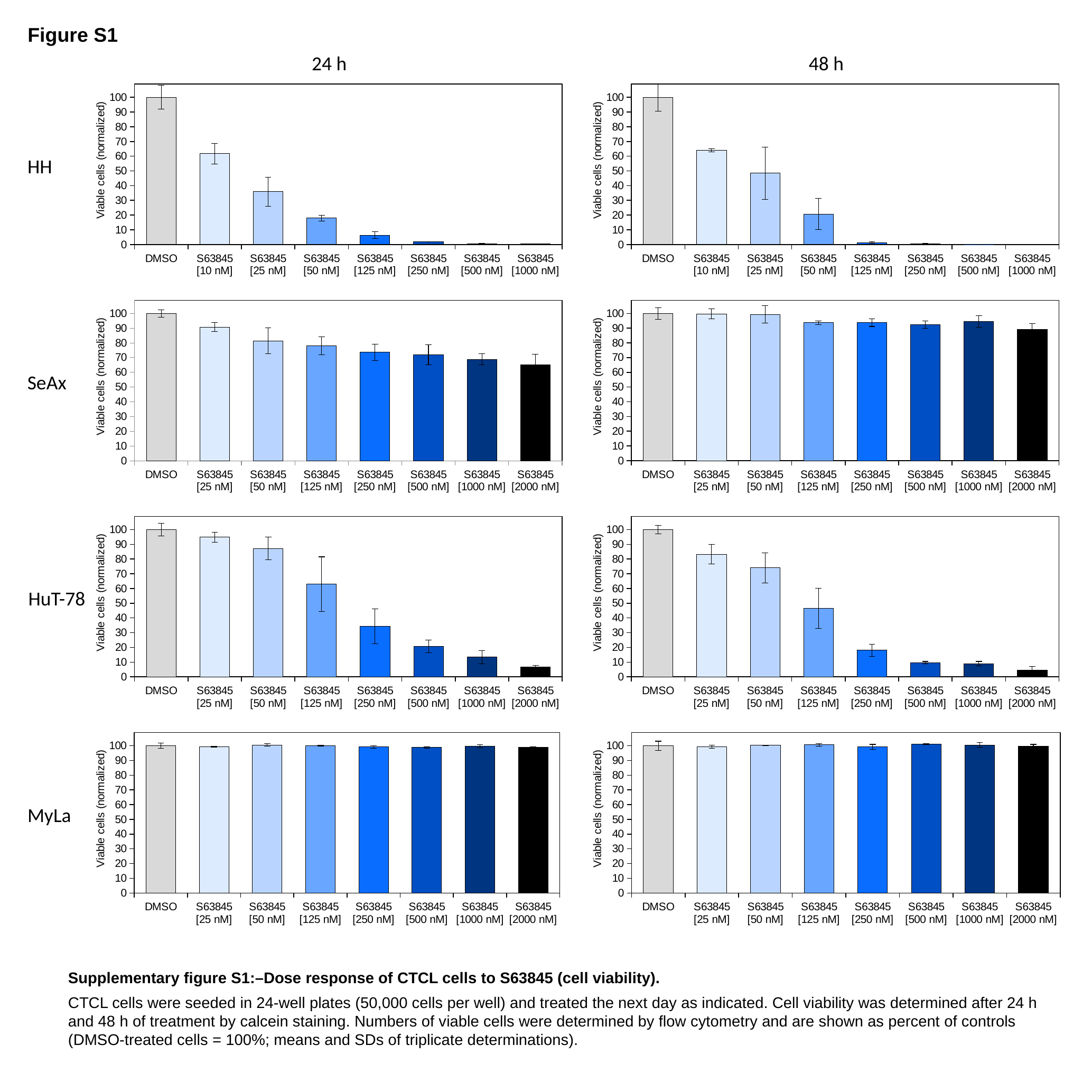

Figure S1
24 h
48 h
### Chart
| Category | |
|---|---|
| DMSO | 100.0 |
| S63845 [10 nM] | 61.70752781077871 |
| S63845 [25 nM] | 35.94880656658386 |
| S63845 [50 nM] | 17.977103358894052 |
| S63845 [125 nM] | 6.399179177016957 |
| S63845 [250 nM] | 1.8684523166648663 |
| S63845 [500 nM] | 0.5238146668106708 |
| S63845 [1000 nM] | 0.3888108867048277 |
### Chart
| Category | |
|---|---|
| DMSO | 100.0 |
| S63845 [10 nM] | 64.01059331877526 |
| S63845 [25 nM] | 48.45049481949542 |
| S63845 [50 nM] | 20.745249268224686 |
| S63845 [125 nM] | 1.375272963806161 |
| S63845 [250 nM] | 0.4971425916461459 |
| S63845 [500 nM] | 0.11150861868698599 |
| S63845 [1000 nM] | 0.05575430934349299 |HH
### Chart
| Category | |
|---|---|
| DMSO | 100.0 |
| S63845 [25 nM] | 90.74511864109972 |
| S63845 [50 nM] | 81.42456877681464 |
| S63845 [125 nM] | 77.96602749321426 |
| S63845 [250 nM] | 73.61001663602137 |
| S63845 [500 nM] | 71.96830400140092 |
| S63845 [1000 nM] | 68.81621574292969 |
| S63845 [2000 nM] | 65.02933193240521 |
### Chart
| Category | |
|---|---|
| DMSO | 100.0 |
| S63845 [25 nM] | 99.73212857828241 |
| S63845 [50 nM] | 99.34831280985127 |
| S63845 [125 nM] | 93.75899568207261 |
| S63845 [250 nM] | 93.77098992483606 |
| S63845 [500 nM] | 92.52758675835598 |
| S63845 [1000 nM] | 94.5266272189349 |
| S63845 [2000 nM] | 89.28914121221813 |SeAx
### Chart
| Category | |
|---|---|
| DMSO | 100.0 |
| S63845 [25 nM] | 94.8726042841037 |
| S63845 [50 nM] | 87.11161217587373 |
| S63845 [125 nM] | 62.872604284103716 |
| S63845 [250 nM] | 34.27733934611049 |
| S63845 [500 nM] | 20.550169109357384 |
| S63845 [1000 nM] | 13.37542277339346 |
| S63845 [2000 nM] | 6.66065388951522 |
### Chart
| Category | |
|---|---|
| DMSO | 99.99999999999999 |
| S63845 [25 nM] | 83.25086396481306 |
| S63845 [50 nM] | 74.01429469054351 |
| S63845 [125 nM] | 46.524505183788875 |
| S63845 [250 nM] | 18.0961357210179 |
| S63845 [500 nM] | 9.68033301916431 |
| S63845 [1000 nM] | 8.989161168708765 |
| S63845 [2000 nM] | 4.394439208294062 |HuT-78
### Chart
| Category | |
|---|---|
| DMSO | 100.00000000000001 |
| S63845 [25 nM] | 99.2902451568661 |
| S63845 [50 nM] | 100.49717126692956 |
| S63845 [125 nM] | 100.0377164409395 |
| S63845 [250 nM] | 99.20452597291275 |
| S63845 [500 nM] | 98.78964512257845 |
| S63845 [1000 nM] | 99.62626435796331 |
| S63845 [2000 nM] | 98.80336019201098 |
### Chart
| Category | |
|---|---|
| DMSO | 100.0 |
| S63845 [25 nM] | 99.24627646146634 |
| S63845 [50 nM] | 100.23080529409644 |
| S63845 [125 nM] | 100.68880954956904 |
| S63845 [250 nM] | 99.25348912690686 |
| S63845 [500 nM] | 101.11435681055934 |
| S63845 [1000 nM] | 100.55898157163979 |
| S63845 [2000 nM] | 99.75837570774281 |MyLa
Supplementary figure S1:–Dose response of CTCL cells to S63845 (cell viability).
CTCL cells were seeded in 24-well plates (50,000 cells per well) and treated the next day as indicated. Cell viability was determined after 24 h and 48 h of treatment by calcein staining. Numbers of viable cells were determined by flow cytometry and are shown as percent of controls (DMSO-treated cells = 100%; means and SDs of triplicate determinations).
