# Supplementary material for: Sensitivity of Cutaneous T-Cell Lymphoma Cells to the Mcl-1 Inhibitor S63845 Correlates with the Lack of Bcl-w Expression
Source: Int J Mol Sci. 2022 Oct 18;23(20):12471. doi: 10.3390/ijms232012471 (PMC9604298; doi:10.3390/ijms232012471)
Supplement: Supplementary file 1 [file ijms-23-12471-s001.zip › CTCL S63 - Figure S2 (dose response - apoptosis) - 02.pptx]

## Slide 1
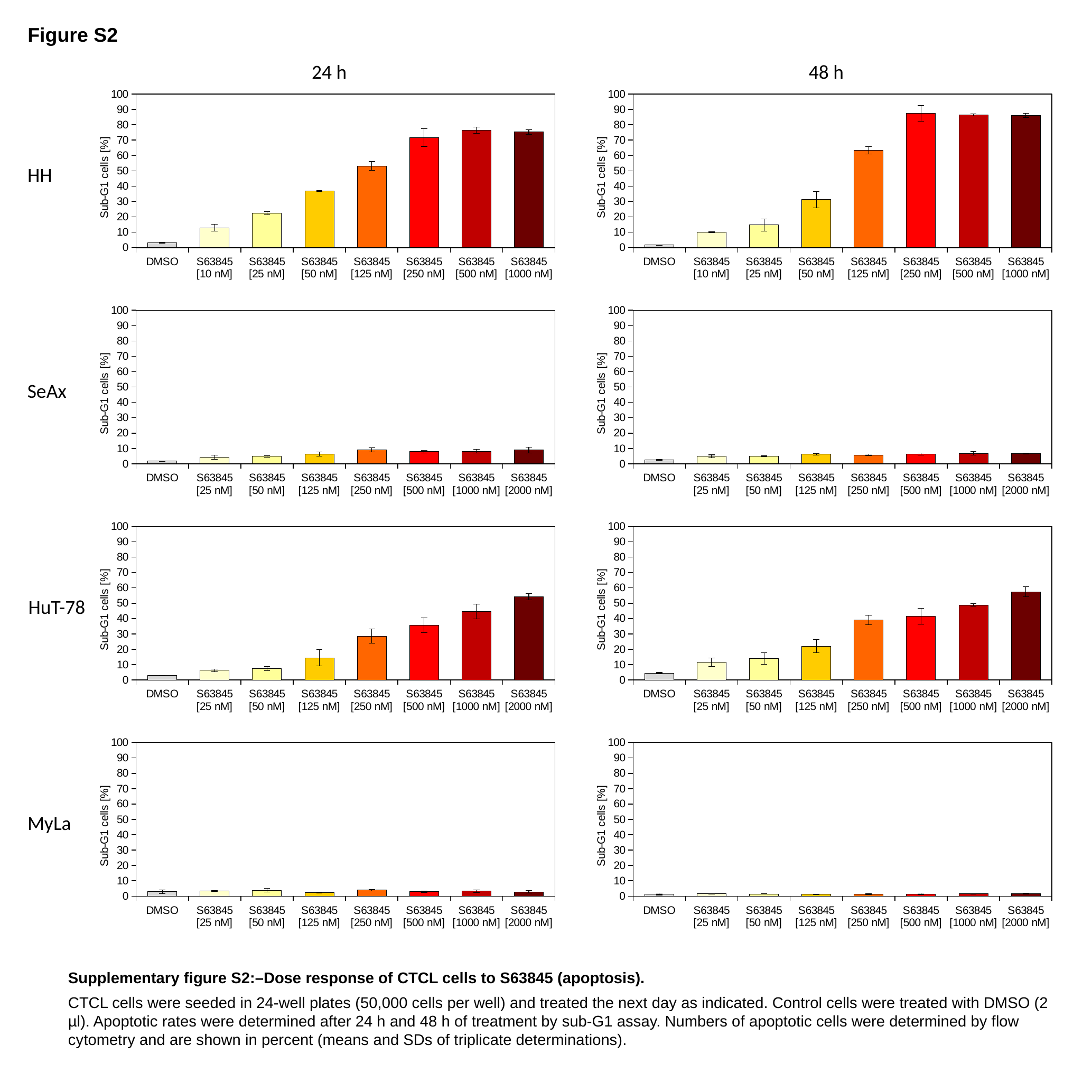

Figure S2
24 h
48 h
### Chart
| Category | |
|---|---|
| DMSO | 3.15 |
| S63845 [10 nM] | 12.923333333333334 |
| S63845 [25 nM] | 22.383333333333336 |
| S63845 [50 nM] | 36.78666666666667 |
| S63845 [125 nM] | 53.123333333333335 |
| S63845 [250 nM] | 71.62333333333333 |
| S63845 [500 nM] | 76.39333333333333 |
| S63845 [1000 nM] | 75.26666666666667 |
### Chart
| Category | |
|---|---|
| DMSO | 1.72 |
| S63845 [10 nM] | 9.99 |
| S63845 [25 nM] | 14.726666666666667 |
| S63845 [50 nM] | 31.22 |
| S63845 [125 nM] | 63.29666666666666 |
| S63845 [250 nM] | 87.31333333333335 |
| S63845 [500 nM] | 86.47333333333334 |
| S63845 [1000 nM] | 85.96666666666665 |HH
### Chart
| Category | |
|---|---|
| DMSO | 1.7833333333333332 |
| S63845 [25 nM] | 4.303333333333334 |
| S63845 [50 nM] | 4.806666666666666 |
| S63845 [125 nM] | 6.329999999999999 |
| S63845 [250 nM] | 9.06 |
| S63845 [500 nM] | 7.916666666666667 |
| S63845 [1000 nM] | 8.216666666666667 |
| S63845 [2000 nM] | 8.986666666666666 |
### Chart
| Category | |
|---|---|
| DMSO | 2.7166666666666663 |
| S63845 [25 nM] | 4.8066666666666675 |
| S63845 [50 nM] | 5.036666666666666 |
| S63845 [125 nM] | 6.206666666666667 |
| S63845 [250 nM] | 5.78 |
| S63845 [500 nM] | 6.3999999999999995 |
| S63845 [1000 nM] | 6.853333333333334 |
| S63845 [2000 nM] | 6.669999999999999 |SeAx
### Chart
| Category | |
|---|---|
| DMSO | 2.9166666666666665 |
| S63845 [25 nM] | 6.276666666666666 |
| S63845 [50 nM] | 7.383333333333333 |
| S63845 [125 nM] | 14.483333333333334 |
| S63845 [250 nM] | 28.486666666666665 |
| S63845 [500 nM] | 35.67666666666667 |
| S63845 [1000 nM] | 44.626666666666665 |
| S63845 [2000 nM] | 54.223333333333336 |
### Chart
| Category | |
|---|---|
| DMSO | 4.483333333333333 |
| S63845 [25 nM] | 11.57 |
| S63845 [50 nM] | 14.106666666666667 |
| S63845 [125 nM] | 21.996666666666666 |
| S63845 [250 nM] | 39.193333333333335 |
| S63845 [500 nM] | 41.60333333333333 |
| S63845 [1000 nM] | 48.836666666666666 |
| S63845 [2000 nM] | 57.48666666666667 |HuT-78
### Chart
| Category | |
|---|---|
| DMSO | 2.9499999999999997 |
| S63845 [25 nM] | 3.483333333333333 |
| S63845 [50 nM] | 3.8333333333333326 |
| S63845 [125 nM] | 2.203333333333333 |
| S63845 [250 nM] | 3.9 |
| S63845 [500 nM] | 2.956666666666667 |
| S63845 [1000 nM] | 3.2899999999999996 |
| S63845 [2000 nM] | 2.776666666666667 |
### Chart
| Category | |
|---|---|
| DMSO | 1.3533333333333335 |
| S63845 [25 nM] | 1.4933333333333334 |
| S63845 [50 nM] | 1.3966666666666667 |
| S63845 [125 nM] | 1.2733333333333334 |
| S63845 [250 nM] | 1.2699999999999998 |
| S63845 [500 nM] | 1.4766666666666666 |
| S63845 [1000 nM] | 1.5566666666666666 |
| S63845 [2000 nM] | 1.5466666666666666 |MyLa
Supplementary figure S2:–Dose response of CTCL cells to S63845 (apoptosis).
CTCL cells were seeded in 24-well plates (50,000 cells per well) and treated the next day as indicated. Control cells were treated with DMSO (2 µl). Apoptotic rates were determined after 24 h and 48 h of treatment by sub-G1 assay. Numbers of apoptotic cells were determined by flow cytometry and are shown in percent (means and SDs of triplicate determinations).
