# Supplementary material for: Sensitivity of Cutaneous T-Cell Lymphoma Cells to the Mcl-1 Inhibitor S63845 Correlates with the Lack of Bcl-w Expression
Source: Int J Mol Sci. 2022 Oct 18;23(20):12471. doi: 10.3390/ijms232012471 (PMC9604298; doi:10.3390/ijms232012471)
Supplement: Supplementary file 1 [file ijms-23-12471-s001.zip › CTCL S63 - Figure S3 (Combinations) - 03.pptx]

## Slide 1
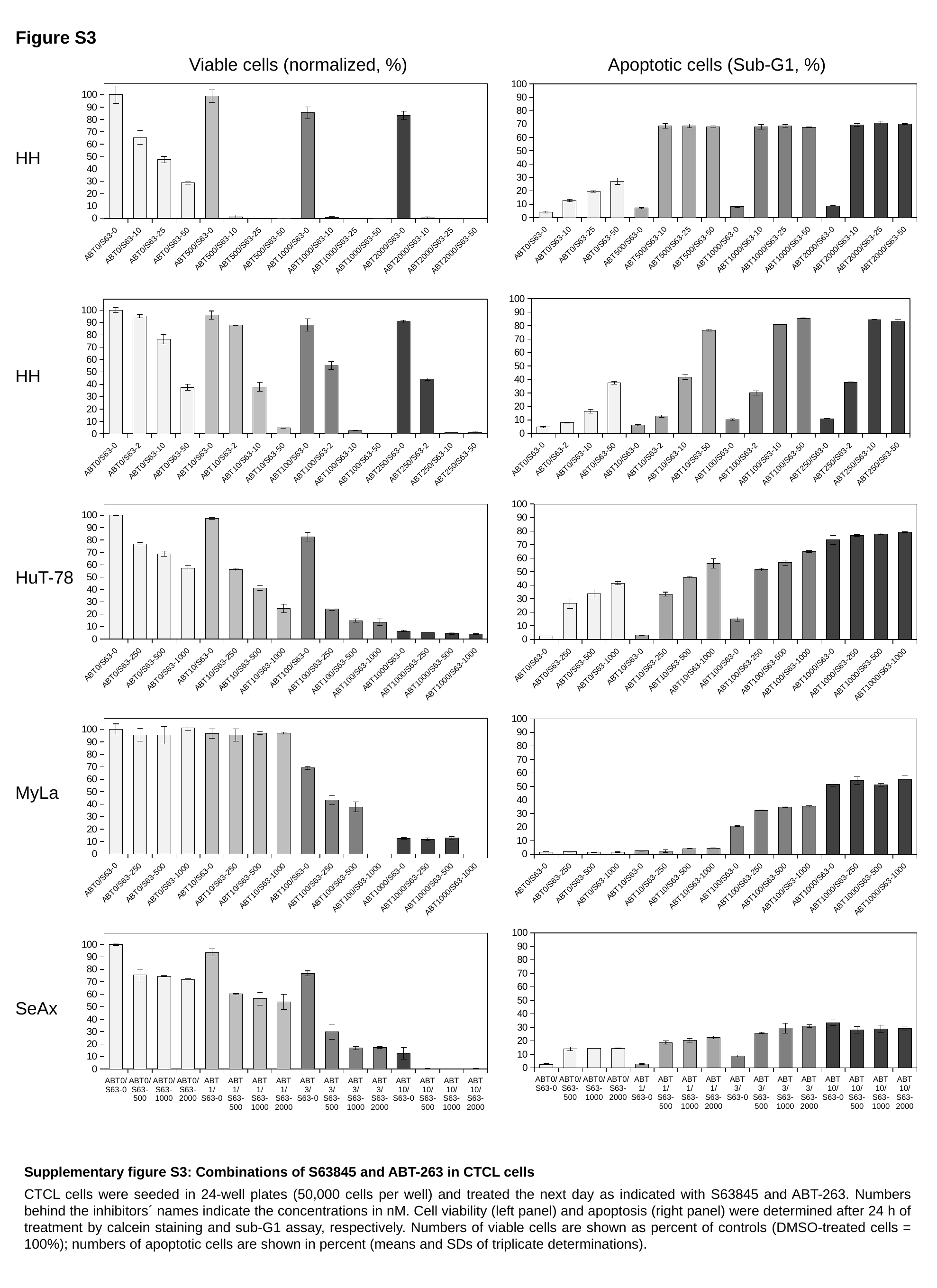

Figure S3
Viable cells (normalized, %)
Apoptotic cells (Sub-G1, %)
### Chart
| Category | |
|---|---|
| ABT0/S63-0 | 100.0 |
| ABT0/S63-10 | 65.43144830816064 |
| ABT0/S63-25 | 47.547125629317414 |
| ABT0/S63-50 | 28.696873902353357 |
| ABT500/S63-0 | 98.82332279592553 |
| ABT500/S63-10 | 1.2118018967334037 |
| ABT500/S63-25 | 0.08781173164734808 |
| ABT500/S63-50 | 0.046832923545252314 |
| ABT1000/S63-0 | 85.39983608476759 |
| ABT1000/S63-10 | 0.6732232759630019 |
| ABT1000/S63-25 | 0.07024938531787847 |
| ABT1000/S63-50 | 0.023416461772626157 |
| ABT2000/S63-0 | 83.245521601686 |
| ABT2000/S63-10 | 0.5795574288724975 |
| ABT2000/S63-25 | 0.1053740779768177 |
| ABT2000/S63-50 | 0.040978808102095775 |
### Chart
| Category | |
|---|---|
| ABT0/S63-0 | 4.0633333333333335 |
| ABT0/S63-10 | 12.823333333333332 |
| ABT0/S63-25 | 19.62333333333333 |
| ABT0/S63-50 | 27.253333333333334 |
| ABT500/S63-0 | 7.186666666666667 |
| ABT500/S63-10 | 68.49 |
| ABT500/S63-25 | 68.76666666666667 |
| ABT500/S63-50 | 67.91333333333334 |
| ABT1000/S63-0 | 8.253333333333332 |
| ABT1000/S63-10 | 68.02333333333333 |
| ABT1000/S63-25 | 68.49 |
| ABT1000/S63-50 | 67.75999999999999 |
| ABT2000/S63-0 | 8.87 |
| ABT2000/S63-10 | 69.33 |
| ABT2000/S63-25 | 70.81333333333333 |
| ABT2000/S63-50 | 70.11666666666667 |HH
### Chart
| Category | |
|---|---|
| ABT0/S63-0 | 100.0 |
| ABT0/S63-2 | 95.14919482159773 |
| ABT0/S63-10 | 76.55115251026209 |
| ABT0/S63-50 | 37.5947268708557 |
| ABT10/S63-0 | 96.0333122829176 |
| ABT10/S63-2 | 88.0131038838017 |
| ABT10/S63-10 | 37.969687401326176 |
| ABT10/S63-50 | 4.64161667192927 |
| ABT100/S63-0 | 87.91837701294601 |
| ABT100/S63-2 | 55.16656141458794 |
| ABT100/S63-10 | 2.6641932428165456 |
| ABT100/S63-50 | 0.0473634354278497 |
| ABT250/S63-0 | 90.85096305652036 |
| ABT250/S63-2 | 44.24534259551626 |
| ABT250/S63-10 | 0.8564887906536155 |
| ABT250/S63-50 | 1.049889485317335 |
### Chart
| Category | |
|---|---|
| ABT0/S63-0 | 4.666666666666667 |
| ABT0/S63-2 | 7.96 |
| ABT0/S63-10 | 16.366666666666667 |
| ABT0/S63-50 | 37.56333333333333 |
| ABT10/S63-0 | 6.1499999999999995 |
| ABT10/S63-2 | 12.75 |
| ABT10/S63-10 | 41.77 |
| ABT10/S63-50 | 76.66333333333334 |
| ABT100/S63-0 | 10.203333333333333 |
| ABT100/S63-2 | 30.05 |
| ABT100/S63-10 | 81.03666666666668 |
| ABT100/S63-50 | 85.56666666666666 |
| ABT250/S63-0 | 10.876666666666667 |
| ABT250/S63-2 | 38.050000000000004 |
| ABT250/S63-10 | 84.51 |
| ABT250/S63-50 | 82.91 |HH
### Chart
| Category | |
|---|---|
| ABT0/S63-0 | 100.0 |
| ABT0/S63-250 | 76.89552666451775 |
| ABT0/S63-500 | 68.90870670821245 |
| ABT0/S63-1000 | 57.39049460979191 |
| ABT10/S63-0 | 97.550231008918 |
| ABT10/S63-250 | 56.036674904193966 |
| ABT10/S63-500 | 41.10167973926435 |
| ABT10/S63-1000 | 24.676766591454463 |
| ABT100/S63-0 | 82.57942050786146 |
| ABT100/S63-250 | 24.1466996167759 |
| ABT100/S63-500 | 14.820386089323447 |
| ABT100/S63-1000 | 13.581175459331684 |
| ABT1000/S63-0 | 6.414526700333082 |
| ABT1000/S63-250 | 4.9675871208051285 |
| ABT1000/S63-500 | 4.369471007485404 |
| ABT1000/S63-1000 | 3.767773360552989 |
### Chart
| Category | |
|---|---|
| ABT0/S63-0 | 2.526666666666667 |
| ABT0/S63-250 | 26.636666666666667 |
| ABT0/S63-500 | 33.836666666666666 |
| ABT0/S63-1000 | 41.593333333333334 |
| ABT10/S63-0 | 3.3966666666666665 |
| ABT10/S63-250 | 33.36666666666667 |
| ABT10/S63-500 | 45.61000000000001 |
| ABT10/S63-1000 | 56.00666666666667 |
| ABT100/S63-0 | 15.003333333333332 |
| ABT100/S63-250 | 51.446666666666665 |
| ABT100/S63-500 | 56.663333333333334 |
| ABT100/S63-1000 | 64.87333333333332 |
| ABT1000/S63-0 | 73.50999999999999 |
| ABT1000/S63-250 | 76.73 |
| ABT1000/S63-500 | 77.99999999999999 |
| ABT1000/S63-1000 | 79.12333333333333 |HuT-78
### Chart
| Category | |
|---|---|
| ABT0/S63-0 | 100.0 |
| ABT0/S63-250 | 95.56257576314145 |
| ABT0/S63-500 | 95.31278698159645 |
| ABT0/S63-1000 | 101.02486867722145 |
| ABT10/S63-0 | 96.60581126253534 |
| ABT10/S63-250 | 95.4744150167138 |
| ABT10/S63-500 | 97.05763508797706 |
| ABT10/S63-1000 | 96.92539396833558 |
| ABT100/S63-0 | 69.07761819050067 |
| ABT100/S63-250 | 43.18774565624655 |
| ABT100/S63-500 | 37.74749292877346 |
| ABT100/S63-1000 | 0.0 |
| ABT1000/S63-0 | 12.518825992726738 |
| ABT1000/S63-250 | 11.809866656871028 |
| ABT1000/S63-500 | 12.698820850016531 |
| ABT1000/S63-1000 | 0.0 |
### Chart
| Category | |
|---|---|
| ABT0/S63-0 | 1.5766666666666669 |
| ABT0/S63-250 | 1.75 |
| ABT0/S63-500 | 1.3933333333333333 |
| ABT0/S63-1000 | 1.5666666666666667 |
| ABT10/S63-0 | 2.4499999999999997 |
| ABT10/S63-250 | 2.16 |
| ABT10/S63-500 | 4.0633333333333335 |
| ABT10/S63-1000 | 4.38 |
| ABT100/S63-0 | 20.726666666666667 |
| ABT100/S63-250 | 32.24 |
| ABT100/S63-500 | 34.93333333333334 |
| ABT100/S63-1000 | 35.343333333333334 |
| ABT1000/S63-0 | 51.74333333333333 |
| ABT1000/S63-250 | 54.45000000000001 |
| ABT1000/S63-500 | 51.25666666666667 |
| ABT1000/S63-1000 | 55.223333333333336 |MyLa
### Chart
| Category | |
|---|---|
| ABT0/S63-0 | 2.606666666666666 |
| ABT0/S63-500 | 14.055 |
| ABT0/S63-1000 | 14.476666666666667 |
| ABT0/S63-2000 | 14.483333333333334 |
| ABT 1/S63-0 | 2.8366666666666664 |
| ABT 1/S63-500 | 18.746666666666666 |
| ABT 1/S63-1000 | 20.39 |
| ABT 1/S63-2000 | 22.373333333333335 |
| ABT 3/S63-0 | 8.806666666666667 |
| ABT 3/S63-500 | 25.655 |
| ABT 3/S63-1000 | 29.30666666666667 |
| ABT 3/S63-2000 | 30.80666666666667 |
| ABT 10/S63-0 | 33.336666666666666 |
| ABT 10/S63-500 | 28.043333333333333 |
| ABT 10/S63-1000 | 28.74333333333333 |
| ABT 10/S63-2000 | 29.146666666666665 |
### Chart
| Category | |
|---|---|
| ABT0/S63-0 | 100.0 |
| ABT0/S63-500 | 75.43532877068353 |
| ABT0/S63-1000 | 74.55167634063935 |
| ABT0/S63-2000 | 71.59317335181494 |
| ABT 1/S63-0 | 93.59785151173872 |
| ABT 1/S63-500 | 60.23564064801178 |
| ABT 1/S63-1000 | 56.41081174737937 |
| ABT 1/S63-2000 | 53.79883912327818 |
| ABT 3/S63-0 | 76.86476652516677 |
| ABT 3/S63-500 | 29.881746513038202 |
| ABT 3/S63-1000 | 16.884692021138353 |
| ABT 3/S63-2000 | 17.343844754396603 |
| ABT 10/S63-0 | 12.41661613098848 |
| ABT 10/S63-500 | 0.3205405873689682 |
| ABT 10/S63-1000 | 0.11695399809408301 |
| ABT 10/S63-2000 | 0.26856103266048686 |SeAx
Supplementary figure S3: Combinations of S63845 and ABT-263 in CTCL cells
CTCL cells were seeded in 24-well plates (50,000 cells per well) and treated the next day as indicated with S63845 and ABT-263. Numbers behind the inhibitors´ names indicate the concentrations in nM. Cell viability (left panel) and apoptosis (right panel) were determined after 24 h of treatment by calcein staining and sub-G1 assay, respectively. Numbers of viable cells are shown as percent of controls (DMSO-treated cells = 100%); numbers of apoptotic cells are shown in percent (means and SDs of triplicate determinations).
