# Supplementary material for: Sensitivity of Cutaneous T-Cell Lymphoma Cells to the Mcl-1 Inhibitor S63845 Correlates with the Lack of Bcl-w Expression
Source: Int J Mol Sci. 2022 Oct 18;23(20):12471. doi: 10.3390/ijms232012471 (PMC9604298; doi:10.3390/ijms232012471)
Supplement: Supplementary file 1 [file ijms-23-12471-s001.zip › CTCL S63 - Figure S4 (Synergy) - 03.pptx]

## Slide 1
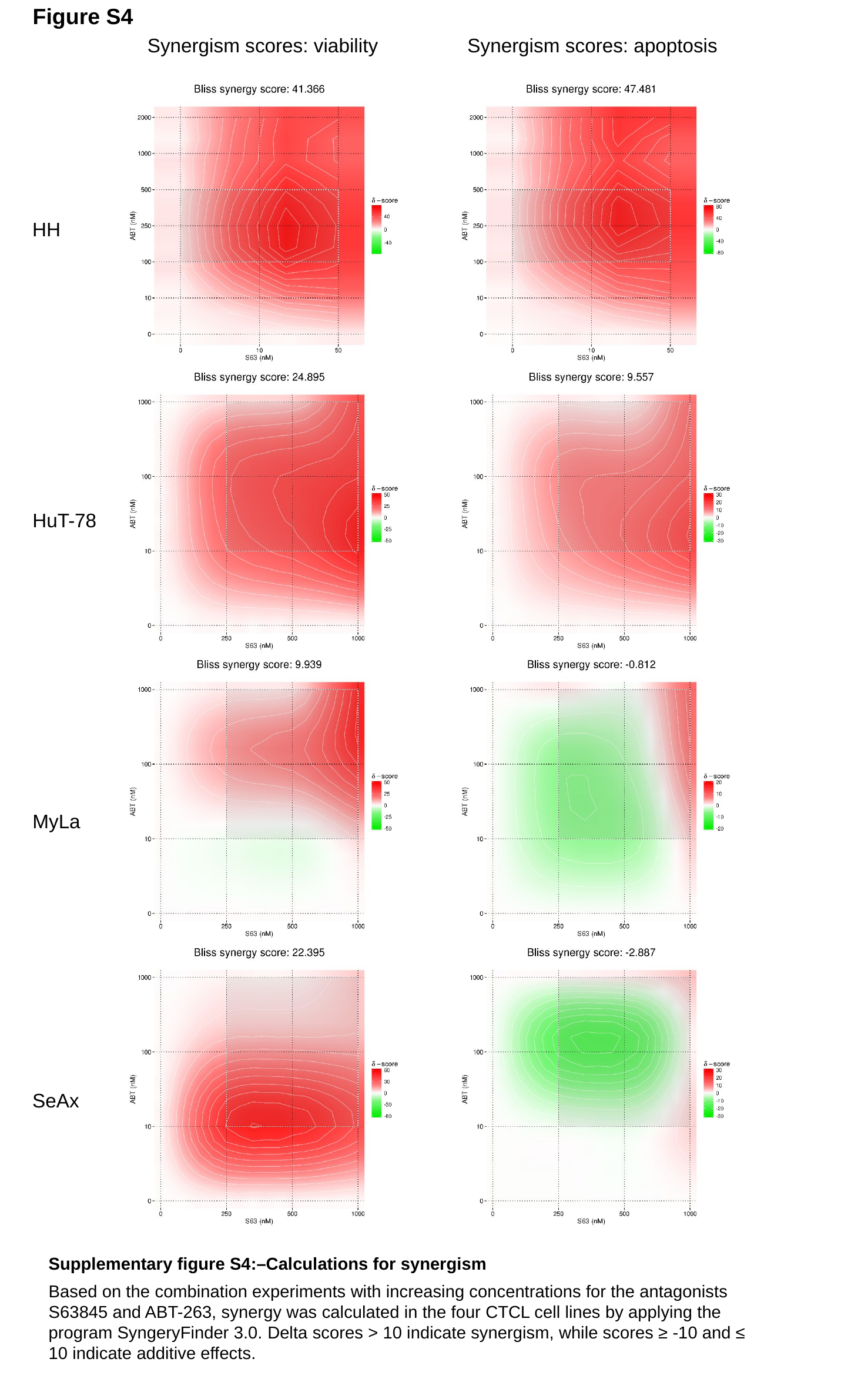

Figure S4
Synergism scores: viability
Synergism scores: apoptosis
HH
HuT-78
MyLa
SeAx
Supplementary figure S4:–Calculations for synergism
Based on the combination experiments with increasing concentrations for the antagonists S63845 and ABT-263, synergy was calculated in the four CTCL cell lines by applying the program SyngeryFinder 3.0. Delta scores > 10 indicate synergism, while scores ≥ -10 and ≤ 10 indicate additive effects.
