# Supplementary material for: Sensitivity of Cutaneous T-Cell Lymphoma Cells to the Mcl-1 Inhibitor S63845 Correlates with the Lack of Bcl-w Expression
Source: Int J Mol Sci. 2022 Oct 18;23(20):12471. doi: 10.3390/ijms232012471 (PMC9604298; doi:10.3390/ijms232012471)
Supplement: Supplementary file 1 [file ijms-23-12471-s001.zip › CTCL S63 - Figure S5 (PBMCs) - 02.pptx]

## Slide 1
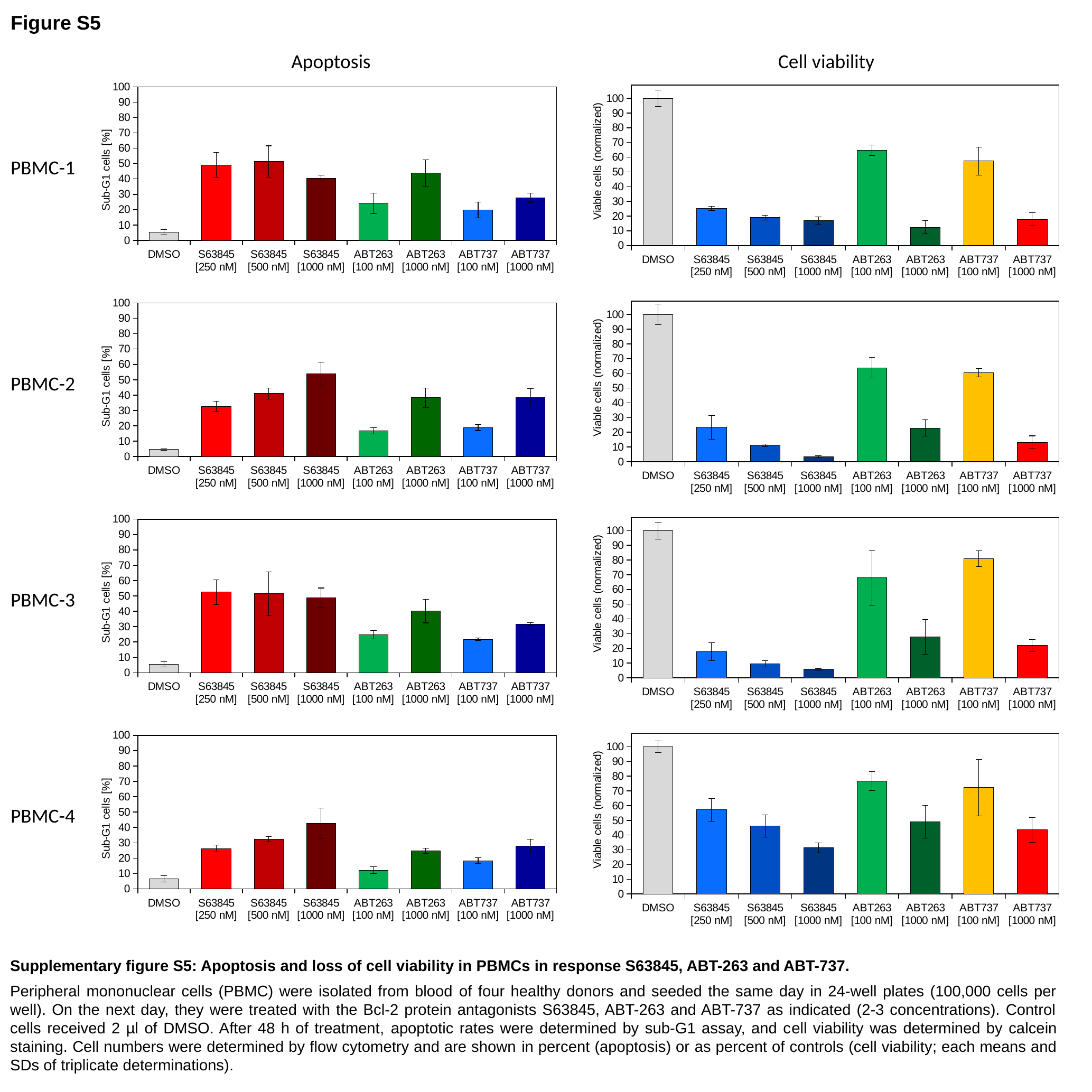

Figure S5
Apoptosis
Cell viability
### Chart
| Category | |
|---|---|
| DMSO | 5.406666666666666 |
| S63845 [250 nM] | 49.11333333333334 |
| S63845 [500 nM] | 51.38666666666666 |
| S63845 [1000 nM] | 40.51333333333333 |
| ABT263 [100 nM] | 24.16 |
| ABT263 [1000 nM] | 43.86333333333334 |
| ABT737 [100 nM] | 19.896666666666665 |
| ABT737 [1000 nM] | 27.55333333333333 |
### Chart
| Category | |
|---|---|
| DMSO | 100.0 |
| S63845 [250 nM] | 25.23783349541246 |
| S63845 [500 nM] | 19.0351359350556 |
| S63845 [1000 nM] | 16.861866305864446 |
| ABT263 [100 nM] | 64.77950192380872 |
| ABT263 [1000 nM] | 12.401166969684157 |
| ABT737 [100 nM] | 57.37600947105832 |
| ABT737 [1000 nM] | 17.84068326920638 |PBMC-1
### Chart
| Category | |
|---|---|
| DMSO | 100.00000000000001 |
| S63845 [250 nM] | 23.348431757969404 |
| S63845 [500 nM] | 11.187078027519016 |
| S63845 [1000 nM] | 3.5424322707460902 |
| ABT263 [100 nM] | 63.81505854200497 |
| ABT263 [1000 nM] | 22.921117853174945 |
| ABT737 [100 nM] | 60.4478249722246 |
| ABT737 [1000 nM] | 13.09717118195026 |
### Chart
| Category | |
|---|---|
| DMSO | 4.553333333333333 |
| S63845 [250 nM] | 32.70666666666667 |
| S63845 [500 nM] | 41.01666666666667 |
| S63845 [1000 nM] | 53.74 |
| ABT263 [100 nM] | 16.680000000000003 |
| ABT263 [1000 nM] | 38.263333333333335 |
| ABT737 [100 nM] | 18.826666666666668 |
| ABT737 [1000 nM] | 38.556666666666665 |PBMC-2
### Chart
| Category | |
|---|---|
| DMSO | 100.0 |
| S63845 [250 nM] | 17.630254387158207 |
| S63845 [500 nM] | 9.517979413231759 |
| S63845 [1000 nM] | 5.822337096993606 |
| ABT263 [100 nM] | 67.87738629664898 |
| ABT263 [1000 nM] | 27.769464471953928 |
| ABT737 [100 nM] | 80.89148868634653 |
| ABT737 [1000 nM] | 22.046887044846503 |
### Chart
| Category | |
|---|---|
| DMSO | 5.44 |
| S63845 [250 nM] | 52.526666666666664 |
| S63845 [500 nM] | 51.39666666666667 |
| S63845 [1000 nM] | 48.73 |
| ABT263 [100 nM] | 24.74333333333333 |
| ABT263 [1000 nM] | 40.1 |
| ABT737 [100 nM] | 21.77333333333333 |
| ABT737 [1000 nM] | 31.76 |PBMC-3
### Chart
| Category | |
|---|---|
| DMSO | 100.00000000000001 |
| S63845 [250 nM] | 57.19569746184262 |
| S63845 [500 nM] | 46.15875175375197 |
| S63845 [1000 nM] | 31.329450278474557 |
| ABT263 [100 nM] | 76.62514348879726 |
| ABT263 [1000 nM] | 49.037030738489015 |
| ABT737 [100 nM] | 72.22482037328346 |
| ABT737 [1000 nM] | 43.54406700395392 |
### Chart
| Category | |
|---|---|
| DMSO | 6.510000000000001 |
| S63845 [250 nM] | 26.273333333333337 |
| S63845 [500 nM] | 32.34666666666667 |
| S63845 [1000 nM] | 42.77333333333333 |
| ABT263 [100 nM] | 12.173333333333332 |
| ABT263 [1000 nM] | 24.689999999999998 |
| ABT737 [100 nM] | 18.346666666666668 |
| ABT737 [1000 nM] | 27.933333333333334 |PBMC-4
Supplementary figure S5: Apoptosis and loss of cell viability in PBMCs in response S63845, ABT-263 and ABT-737.
Peripheral mononuclear cells (PBMC) were isolated from blood of four healthy donors and seeded the same day in 24-well plates (100,000 cells per well). On the next day, they were treated with the Bcl-2 protein antagonists S63845, ABT-263 and ABT-737 as indicated (2-3 concentrations). Control cells received 2 µl of DMSO. After 48 h of treatment, apoptotic rates were determined by sub-G1 assay, and cell viability was determined by calcein staining. Cell numbers were determined by flow cytometry and are shown in percent (apoptosis) or as percent of controls (cell viability; each means and SDs of triplicate determinations).
